# The protein and mRNA expression levels of glial cell line-derived neurotrophic factor in post stroke depression and major depressive disorder

**DOI:** 10.1038/s41598-017-09000-y

**Published:** 2017-08-17

**Authors:** Yanran Zhang, Haitang Jiang, Yingying Yue, Yingying Yin, Yuqun Zhang, Jinfeng Liang, Shenghua Li, Jun Wang, Jianxin Lu, Deqin Geng, Aiqin Wu, Yonggui Yuan

**Affiliations:** 1 School of Medicine, Southeast University, Nanjing, 210009 P.R. China; 2 Department of Psychosomatics and Psychiatry, ZhongDa Hospital, School of Medicine, Institute of Psychosomatic Medicine, Southeast University, Nanjing, 210009 P.R. China; 3 Department of Neurology, Jiangning Nanjing hospital, Nanjing, 211100 P.R. China; 4 Department of Neurology, The Affiliated Nanjing First hospital of Nanjing Medical University, Nanjing, 210006 PR China; 5 Department of Neurology, The peoples’ hospital of Gaochun county, Nanjing, 211300 P.R. China; 6 Department of Neurology, Affiliated Hospital of Xuzhou Medical College, Xuzhou, 221000 P.R. China; 7 Department of Psychosomatics, The First Affiliated Hospital of Suzhou University, Suzhou, 215006 P.R. China

## Abstract

Previous studies have indicated that the level of glial cell line-derived neurotrophic factor (GDNF) may be correlated with stroke and depression. Here, we investigated whether GDNF can be a discriminant indicator for post stroke depression (PSD). 159 participants were divided into four groups: PSD, stroke without depression (Non-PSD), major depressive disorder (MDD) and normal control (NC) group, and the protein and mRNA expression levels of GDNF in serum were measured. The results showed that only MDD group had statistical difference in protein and mRNA levels compared with the other three groups (Bonferroni test, P < 0.05). The results of receiver operating curve (ROC) analysis supported GDNF as general distinguishing models in PSD and MDD groups with the area under the curve (AUC) at 0.797 (*P* < 0.001) and 0.831 (*P* < 0.001) respectively. In addition, the Spearman analysis demonstrated that the GDNF protein level negatively correlated with the value of Hamilton depression rating scale (HAMD) in PSD patients (correlation coefficient = −0.328, *P* = 0.047). Together, these findings suggest the protein and mRNA expression levels of GDNF decreased in patients with depression. GDNF may serve as a potential biomarker for differential diagnosis of PSD from MDD patients.

## Introduction

Stroke is a major disease that often results in permanent tissue damage due to loss of blood supply to the brain^1^. Stroke is a leading cause of death in the Western countries and in China^2^. Nearly 30% of the stroke survivors are likely to experience post-stroke depression (PSD), an important complication of stroke as well as a subtype of depression, which leads to greater disability and increased mortality. Early antidepressant treatment of PSD has been shown to increase not only the physical and cognitive recovery from stroke, but also survival time^1, 3^. Therefore, early diagnosis of PSD and other subtypes of depression related to stoke including major depressive disorder (MDD) would greatly benefit patients with relevant disorders in terms of decreasing their disability and mortality.

Based on previous preclinical and clinical studies, there are several mechanisms, including neurotrophic signaling, cellular plasticity and activation of the hypothalamic-pituitary-adrenal may participate in the pathogenesis and progression of PSD^4^. As to the neurotrophic and neurochemical factors, structural and functional modification of the brain associated with synaptic plasticity has been suggested as an etiology of psychiatric disorders^5^. The degeneration or dysfunction of glial, especially astrocytes, plays a critical role in the pathogenesis of depression^6^. Astrocytes produce neurotrophic and growth factors that support neurogenesis, gliogenesis, brain development, neural plasticity, and survival^6, 7^. Glial cell line-derived neurotrophic factor (GDNF) is a neurotrophic factor from the transforming growth factor-β superfamily that is extensively distributed in mammalian brains, including hypothalamus, substantia nigra, and thalamus^8^. The major functions of GDNF include developing and maintaining neurons and glial cells, as well as protecting them against oxidative stress^9^. GDNF has also been a potent neurotherapeutic agent for acute ischemic stroke^10, 11^.

Multiple studies have previously investigated the relation between GDNF and the development and treatment of stroke and depression. Five different trials conducted during 1997 to 2010 demonstrated how topical application of GDNF on the cortical surface reduced stroke volume, brain edema formation and cell death in focal brain ischemia in rats^10, 12–15^. In another rat model study, treatment with levodopa enhanced functional recovery after experimental stroke, accompanied with an increase of GDNF level in the ischemic hemisphere, suggesting involvement of GDNF in the mechanisms of tissue reorganization and plasticity^16^. Decreased peripheral levels of GDNF have also been detected in many patients with depression^8, 17–20^. However, other trials have reported contradictory findings. Lee *et al*. has shown in their research that plasma GDNF level in MDD patients at baseline, or to the end of antidepressant treatment, had no significant difference compared with that in healthy controls^21^. Meanwhile, multiple studies have reported significantly increased GDNF in serum and parietal cortex of bipolar depression as well as late-onset depression patients compared with normal controls^22–24^. In addition, increased serum GDNF levels were detected in depressed patients undergone successful antidepressant treatment and electroconvulsive therapy^19, 25, 26^. However, to the best of our knowledge, there is no research that has been reported to investigate the relationship between GDNF and PSD.

In this study, we hypothesized that serum GDNF protein and mRNA levels in PSD patients are significantly lower than that in NC group, and we have for the first time, explored the relationship between serum GDNF and PSD. GDNF may serve as a potential biomarker that contributes to differential diagnosis of PSD from MDD.

## Subjects and Methods

### Study population

Patients aged between 18 and 80 years of age suffered from ischemic stroke or major depressive disorder were enrolled in the study during the period from July 2013 to December 2014. These include patients of PSD (39), Non-PSD (42), and MDD (40). 38 roughly age-matched normal controls (NC) were recruited as well. All individuals included in the study met the standards of right-handed and have signed the informed consent. This study was approved by the Medical Ethics Committee for Clinical Research of Zhongda Hospital Affiliated to Southeast University, and all methods were performed in accordance with the Declaration of Helsinki. The clinical diagnosis of stroke was performed by a neurologist, and confirmed by computed tomography (CT) or magnetic resonance imaging (MRI).

The diagnostic criteria for PSD were as follows^27^: (1) Had stroke history before, or stroke occurs earlier than depressive symptoms; (2) Met at least two depressive symptoms in nine symptoms of MDD in DSM-IV except core criterion symptoms of depressed mood and loss of interest or pleasure; (3) Impairment to fit personal and work functioning; (4) More than one week of depressive symptoms; (5) Free of other major psychiatric disorders, including schizophrenia, bipolar disorder, substance abuse (caffeine, nicotine and alcohol).

A Structured Clinical Interview according to the Diagnostic Statistical Manual of Mental Disorder (DSM-IV) was used to diagnose MDD. All subjects were reviewed by two trained senior psychiatrists. The severity of depression and cognitive function were assessed by both the score of HAMD-17 and the mini mental state examination (MMSE)^27^.

### Blood collection

Venous blood samples of all participants were collected from the antecubital veins to EDTA-anticoagulant and coagulant tubes. Samples in anticoagulant tubes was directly stored at −80 °C for further study, and the ones in coagulant tubes were separated by centrifugation for 30 min at 3000 rcf, then the isolated serum samples were stored at −80 °C, which were ready to be assayed.

### Determination of serum protein level using Enzyme-Linked Immunosorbent Assay (ELISA)

Serum concentrations of GDNF were measured using ELISA kits (ab100525 – GDNF human ELISA kit, abcam, UK) according to the manufacturer’s instructions. The concentrations were expressed as ng of protein/ml.

### Quantitative real-time polymerase chain reaction (qRT-PCR)

According to the manufacturer’s protocol, total RNAs from the peripheral blood lymphocytes were extracted with the QIAamp RNA Blood Mini Kit (Qiagen, Hilden, Germany). After assessing the RNA quality and quantity with NanoDrop, one microgram of total RNA was used for cDNA synthesis using the random hexanucleotide primers and the Sensiscript Reverse Transcription Kit (Qiagen, Hilden, Germany) following the instruction manual. qRT-PCR was performed in triplicate using ViiA7TM sequence detection system (Applied Biosystems, Foster City, CA) by incorporating SybrGreen fluorescent dye, and the primers were designed by the Primer Express Software v2.0 (see Table 1). qRT-PCR was carried out in a final volume of 16ul, including 1ul cDNA, 8 ul 2× SYBGEEN PCR mix, 1ul of each primer and 5ul H2O. Amplification started with denaturation at 95 °C for 2 min followed by 40 cycles at 94 °C for 10 s, 59 °C for 10 s and 72 °C for 40 s with additional extension time of 5 mins at 72 °C. Real-time PCR data were calculated by the data analysis module automatically. Relative expression levels were measured by the 2^−ΔΔCT^ method using glyceraldehyde-3-phosphate dehydrogenase (GAPDH) as endogenous control^27^.

**Table 1:** The primers for qPCR.

Gene name	Primer	Sequence
GDNF	101-GDNF-F	CCAACCCAGAGAATTCCAGA
	101-GDNF-R	AGCCGCTGCAGTACCTAAAA

Note: qRT-PCR: quantitative real-time polymerase chain reaction; GDNF: glial cell line-derived neurotrophic factor.

### Statistical analysis

Mean (M) and standard deviation (SD) were used to describe demographic and clinical characteristics. Continuous variables of general characteristics, clinical and biological changes were described with nonparametric test (Kruskal-Wallis H test) and one-way analysis of variance (ANOVA). The data exceeding M ± 3 SD as outlier were omitted. Receiver operating characteristic (ROC) curve expressed as area under the curve (AUC) with the corresponding 95% confidence interval (CI) was set for the prediction of GDNF to PSD and for the differential function from MDD. SPSS Version 23.0 statistical software (SPSS Inc. Chicago, IL) was used to conduct all the analysis.

## Results

### Demographic and neuropsychological results

In Table 2 the demographic and neuropsychological characteristics are as illustrated. No significant difference was observed in education level among the four groups (*P* = 0.305). However, statistically significant differences were found in age, gender, HAMD and MMSE (all *P* < 0.05).

**Table 2:** Demographic and data, GDNF protein and mRNA expression levels among four groups.

Item	PSD group (*n* = 39)	Non-PSD group (*n* = 42)	MDD group (*n = *40)	NC group (*n* = 38)	F/X2/Z	*P* value
Age (years)	62.44 ± 10.34	61.10 ± 6.58	58.70 ± 9.92	57.58 ± 5.28	2.76	0.044^a^
Gender (male/female)	20/19	25/17	9/31	22/16	14.32	0.003^b^
Education level (years)	8.15 ± 4.36	9.43 ± 4.01	9.40 ± 4.77	8.24 ± 2.56	1.22	0.305^a^
Active smokers n (%)	16 (41.0%)	21 (50.0%)	9 (22.5%)	—	6.80	0.033^b^
Alcohol consumption n (%)	9 (23.1%)	16 (38.1%)	16 (40.0%)	—	3.03	0.219
HAMD	16.00 ± 5.45	3.36 ± 2.02	19.45 ± 4.75	2.18 ± 1.98	203.32	<0.001^a^
MMSE	22.64 ± 6.80	27.00 ± 2.45	27.08 ± 1.45	28.47 ± 1.45	28.46	<0.001^c^
NIHSS	5.64 ± 4.69	2.64 ± 2.90	—	—	−3.44	0.001^d^
GDNF Protein level (ng/ml)^†^	0.46 ± 0.21	0.40 ± 0.18	0.26 ± 0.09	0.40 ± 0.21	22.70	<0.001^c^
GDNF mRNA expression level^‡^	0.19 ± 0.15	0.15 ± 0.13	0.05 ± 0.05	1.00 ± 1.60	29.44	<0.001^c^

Note: Data reported as mean ± SD and ratio. PSD: post stroke depression; Non-PSD: stroke without depression; MDD: major depressive disorder; NC: normal control. HAMD: Hamilton depression rating scale; MMSE: mini mental state examination. NIHSS: National Institutes of Health Stroke Scale. ^a^One-way ANOVA. ^b^Chi square test. ^c^Kruskal-Wallis test. ^d^Mann-Whitney U test. ^†^nine abnormal data were excluded (two PSD patients, two Non-PSD patients, four MDD patients and one NC subject); ^‡^five abnormal data were excluded (four Non-PSD patients and one MDD patient).

Compared with NC, there was a decreased GDNF protein level in MDD (F_Bonferroni_ = −30.798, standard error (SE) = 9.460, *P* = 0.008, 95% CI, −56.100~−5.497). In addition, the GDNF protein level in patients with MDD was lower than that in both PSD (F_Bonferroni_ = −45.758, SE = 9.460, *P* < 0.001, 95% CI, −71.059~−20.456) and non-PSD (F_Bonferroni_ = −36.217, SE = 9.283, *P* = 0.001, 95% CI, −61.046~−11.387) patients. However, PSD, non-PSD and NC groups had no significant statistical differences with each other (see Table 2).

Similar to the protein level examination, compared with NC, the GDNF mRNA level decreased in MDD (F_Bonferroni_ = −43.640, standard error (SE) = 9.227, *P* < 0.001, 95% CI, −68.310~−18.971). Moreover, the GDNF mRNA level was lower in MDD than both PSD (F_Bonferroni_ = −48.487, SE = 9.167, *P* < 0.001, 95% CI, −72.996~−23.978) and non-PSD (F_Bonferroni_ = −43.640, standard error (SE) = 9.227, *P* < 0.001, 95% CI, −68.310~−18.971) patients. PSD, non-PSD, and NC groups had no significant statistical differences with each other (see Table 2).

### The correlations between GDNF and HAMD in PSD patients

**Figure 1:**
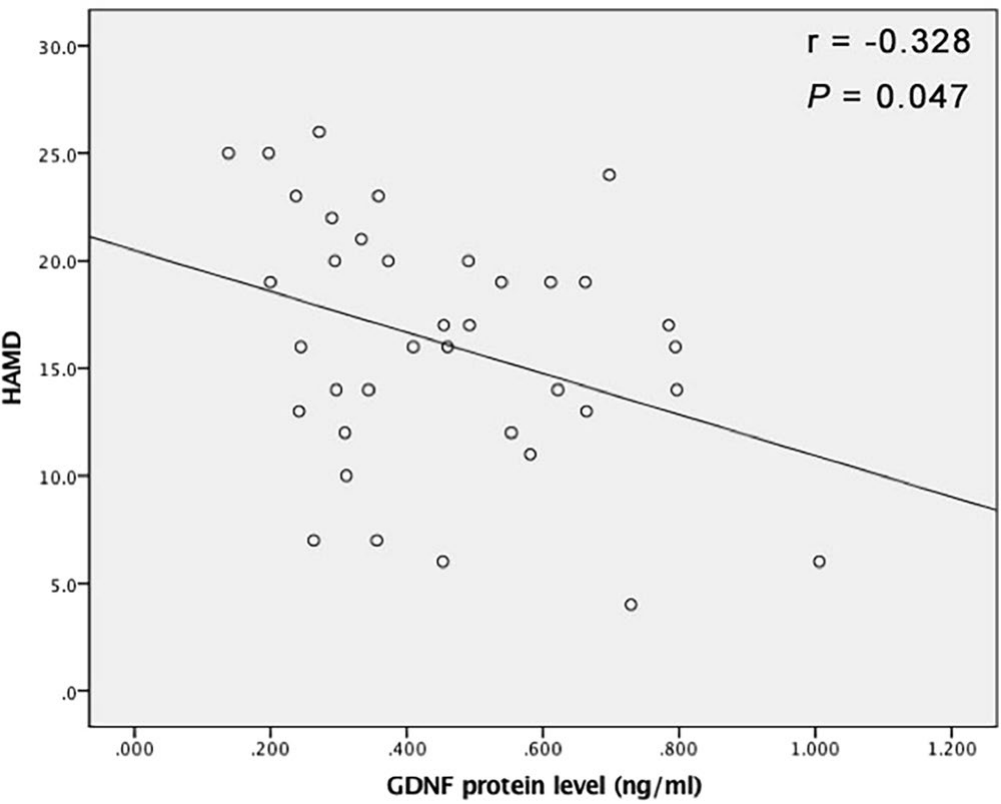
The correlation analysis showed that GDNF protein level negatively correlated with the value of HAMD-17 in PSD patients (correlation coefficient = −0.328, *P* = 0.047). Abbreviations: GDNF, glial cell line-derived neurotrophic factor; HAMD, Hamilton depression rating scale; PSD, post stroke depression.

The spearman analysis showed that GDNF protein level negatively correlated with the value of HAMD-17 in PSD patients (correlation coefficient = −0.328, *P* = 0.047) (see Fig. 1). Nevertheless, no correlations were observed between the protein level of GDNF and the value of HAMD-17 in MDD patients (*P* > 0.05).

### The differentiating function of GDNF in PSD and MDD patients

**Figure 2:**
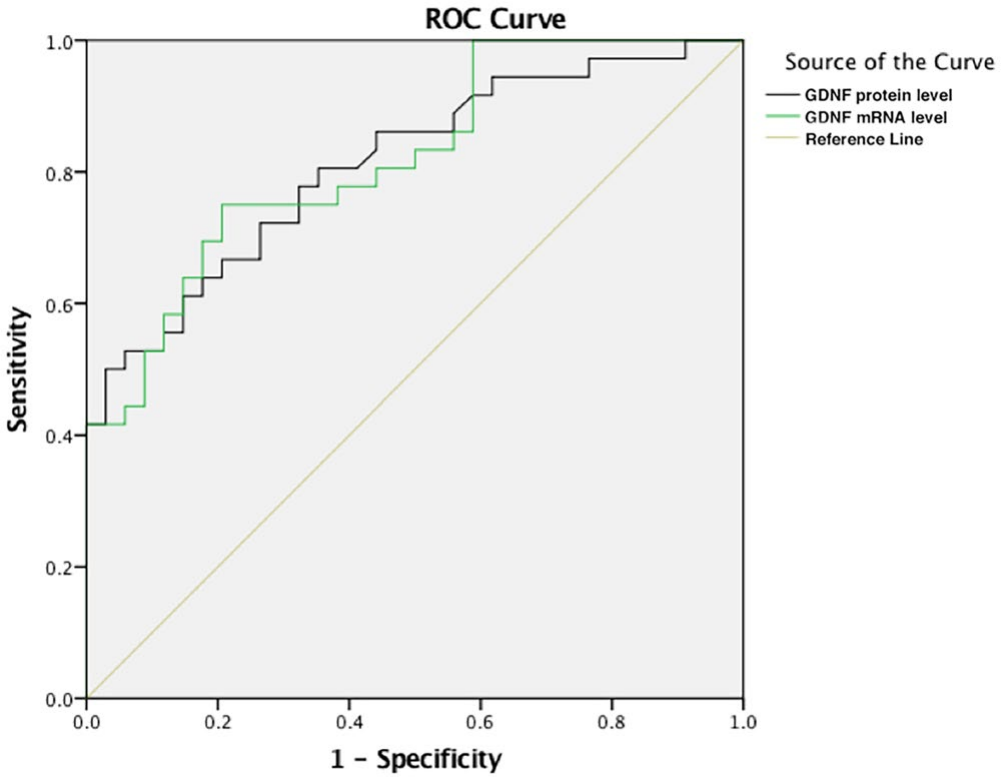
The results of ROC analysis in PSD and MDD groups indicated that the protein and mRNA levels of GDNF could serve as general distinguishing models with the AUC at 0.797 (95% CI, 0.696~0.898, *P* < 0.001) and 0.831 (95% CI, 0.744~0.918, *P* < 0.001) respectively. Abbreviations: ROC, receiver operating curve; PSD, post stroke depression; MDD, major depressive disorder. GDNF, glial cell line-derived neurotrophic factor; AUC, area under the curve.

From the results of receiver operating curve (ROC) analysis in PSD and MDD groups, the protein and mRNA levels of GDNF could serve as general distinguishing models with the AUC at 0.797 (95% CI, 0.696~0.898, *P* < 0.001) and 0.831 (95% CI, 0.744~0.918, *P* < 0.001) respectively (see Fig. 2).

## Discussion

It was previously suggested that low levels of neurotrophic factors (majorly the brain-derived neurotrophic factor, BDNF) contribute to the major depressive episode, while an increase of BDNF ameliorates the depressive symptoms^28^. Recently, multiple studies have investigated the role of GDNF, the Glial cell line-derived neurotrophic factor, in depression and have found that serum GDNF protein levels in MDD patients were significantly decreased compared with that of the control subjects^8^. In our studies, we did detect a significant decrease of serum GDNF protein in MDD patients which was in accordance with previous findings. We further investigated the transcriptional level of GDNF and found that GDNF mRNA was significantly lower in MDD than in NC, PSD and non-PSD groups. We did not detect any decrease of GDNF in PSD group compared with NC group, while neither the protein nor the mRNA level of GDNF, showed significant difference among the groups of NC, PSD, and non-PSD.

The precise mechanisms contributing to the decreased GDNF protein and mRNA levels in depression remain unknown. GDNF is known to play its role through binding to GDNF-family receptors α1 (GFR-α1) and activation of tyrosine kinase (c-Ret) signaling^29^. GDNF may protect dopaminergic and serotonergic neurons from oxidative stress and neuro-inflammatory damage, and also shows its neurotrophic effect on various brain neurons^9, 29^. A previous study demonstrated that overexpression of GDNF in the dorsal CA1 hippocampal astrocytes enhanced local cholinergic, dopaminergic, and serotonergic transmission^9^. We therefore, surmise that the decrease of GDNF in MDD patients attenuated the protective effect for neural cells, leading to increased neuronal damage, and thus the symptoms of MDD. As to the research that showed the opposite result of increased GDNF in depression, a compensatory mechanism may exist which will cause suppression of the oxidative stress^23^.

Given the difference of therapeutic strategy and efficiency, it is of urgent need to find ways of early diagnosis of PSD and differential diagnosis of PSD from other subtypes of depression. In the current study, we have shown that both protein and mRNA levels of GDNF have a significant difference between PSD and MDD groups. The ROC analysis showed the consistent results (protein and mRNA; AUC = 0.797 and 0.831, respectively). Therefore, the level of GDNF might be used as a measurement to distinguish PSD from MDD.

The GDNF level differences between PSD and MDD groups suggest that PSD has different pathological mechanisms with MDD. The stress property (acute or chronic) and the time accumulation impacting the changes of gene expression may be a possible explanation^4^, as MDD patients suffer chronic stress while PSD patients suffer acute stress of stroke and chronic stress of depression as well.

Chronic stress is known as a risk factor of MDD and is able to influence the expression of GDNF. Increased DNA methylation interrelated with histone modification has been shown to contribute to the formation of depression-susceptible phenotype in BALB mice that suffer from chronic ultra-mild stress^25^. In addition, microRNAs may also reduce the expression of GFR-α1a, which is a specific isoform of the GFRA1 gene, in the basolateral amygdala of depressed patients, leading to changes of neuronal responses to GDNF^25^. These results may help to explain the decrease of GDNF in MDD group compared with that in NC group.

Chronic stress leads to a decrease of GDNF, yet acute stress of stroke may lead to the opposite. A possible explanation for non-decreased GDNF in PSD is that transient focal ischemia and reperfusion in stroke stimulated the expression of GDNF and its receptors GFR-α1 and c-Ret^30^, and this may counteract the descent of GDNF in the chronic stress caused by depression.

In addition, there were studies that focused on how depression be diagnosed in patients with stroke. It has been pointed out that the depressive symptoms in patients with stroke were very similar to those described in Diagnostic and Statistical Manual of Mental Disorders, 3rd Edition (DSM-III) or 4th Edition (DSM-IV), suggesting that PSD might be the same disease as depression^31–33^. However, our findings indicated that the changes in GDNF were associated with the trait, rather than the stage, of MDD, supporting that MDD and PSD are biologically different.

## Limitations

The present research still had limitations which need to be optimized in future studies. First, the number of participants in current study was relatively small, larger sample size in future studies may help with the conclusions drawn from this study. We also notice that even the confounders like gender, age and education level were considered in the statistical analysis, the age and gender of the four groups were not matched perfectly due in part to the limited sample population.

## Conclusions

We have shown that the protein and mRNA expression levels of GDNF decreased in patients with depression. The severity of depression in PSD positively correlated with the degrading of GDNF. Furthermore, the changes in GDNF were associated with the trait, rather than the stage, of MDD, suggesting that MDD and PSD are biologically different. The significantly decreased levels of GDNF in MDD compared with PSD may help with the differential diagnosis of PSD from MDD. Finally, as no significant difference of GDNF levels between PSD and non-PSD (protein and mRNA, AUC = 0.573, *P* = 0.273; AUC = 0.563, *P* = 0.338, respectively), it is therefore, hard to predict whether PSD would supervene upon stroke.
